# Secondary Angio-Embolization After Emergent Pelvic Stabilization and Pelvic Packing Is a Safe Option for Patients With Persistent Hemorrhage From Unstable Pelvic Ring Injuries

**DOI:** 10.3389/fsurg.2020.601140

**Published:** 2020-12-17

**Authors:** Thomas Lustenberger, Philipp Störmann, Kathrin Eichler, Christoph Nau, Maren Janko, Ingo Marzi

**Affiliations:** ^1^Department of Trauma, Hand and Reconstructive Surgery, Hospital of the Goethe University Frankfurt am Main, Frankfurt, Germany; ^2^Institute for Diagnostic and Interventional Radiology, Hospital of the Goethe University Frankfurt am Main, Frankfurt, Germany

**Keywords:** pelvic ring fracture, management, hemodynamic instability, pelvic packing, angiography, embolization, external fixation

## Abstract

**Introduction:** In patients with severe pelvic ring injuries, exsanguination still is the leading cause of death in the early post-injury phase. While mechanical pelvic ring stabilization and pre-peritoneal pelvic packing are mainly addressing venous bleeding, angio-embolization aims to control arterial bleeding. The goal of the present study was to evaluate the rate of postoperative angio-embolization after mechanical pelvic ring injury stabilization and pre-peritoneal pelvic packing. Bleeding sources detected in the angiography and the patient's outcome were investigated.

**Patients and Methods:** Retrospective observational cohort study at a single academic level I trauma center, reviewing all patients with pelvic ring injuries admitted from 01/2010 to 12/2019. Patients with emergent mechanical pelvic ring stabilization (supraacetabular external fixator and/or pelvic C-clamp) and direct pre-peritoneal pelvic packing were further analyzed. Patients that underwent postoperative angio-embolization were compared with those that did not. All postoperative angio-embolizations were evaluated with regards to bleeding sources and type of embolization.

**Results:** During the study period, a total of 39 patients required immediate mechanical pelvic stabilization and direct pre-peritoneal pelvic packing. Of these, 12 patients (30.8%) underwent a postoperative angio-embolization. The following vessels were identified as bleeding sources: superior gluteal artery (*n* = 6), obturator artery (*n* = 2), internal pudendal artery (*n* = 2), unnamed branches of the internal iliac artery (*n* = 3). A selective embolization was successful in 11 patients; in 1 patient, an unilateral complete occlusion of the internal iliac artery was performed to control the bleeding. Mean time from hospital admission to the surgical procedure was 52.8 ± 14.7 min and the mean time from admission to angio-embolization was 189.1 ± 55.5 min. The in-hospital mortality rate of patients with angio-embolization was 25.0% (*n* = 3). Of these, 2 patients died due to multiple organ failure and 1 patient due to severe head injury.

**Conclusion:** Secondary angio-embolization after external pelvic fixation and pre-peritoneal pelvic packing was effective in controlling ongoing bleeding. The most frequently detected bleeding vessel was the superior gluteal artery, which is difficult to surgically address, further highlighting the importance of angio-embolization in the management algorithm.

## Introduction

Severe fractures of the pelvic ring pose significant challenges to the entire trauma team in terms of life-threat and functional outcome. Management algorithms focusing on volume resuscitation, mechanical stabilization of the pelvic ring injury, and coagulation management have improved over the last years and have resulted in significant achievements in the treatment of these usually polytraumatized patients (1). Nevertheless, the overall mortality rate in these patients remains high, ranging from 5 to 10% for patients with any type of pelvic fracture (2–4), up to 60–70% for hemodynamically compromised pelvic fracture patients or patients with open pelvic ring injuries (5–8).

In the past years, many different approaches to effectively manage the hemodynamically unstable patient with pelvic ring injury have been suggested and have been lively and controversially discussed in the contemporary literature (2, 9–25). These controversies have their origin—among other reasons—in different trauma system developments comparing European countries and North America and therefore have resulted in different favored pathways in the treatment of these highly challenging injuries. In the Anglo-American area, arteriography has become increasingly available over the last decade and has subsequently been implemented as the first line treatment even in the hemodynamically unstable pelvic trauma patient. In European countries, however, many trauma surgeons were trained in orthopedic surgery and are therefore highly familiar with early pelvic stabilization techniques which can easily be combined with pelvic packing in the initial phase. Therefore, two different fundamental treatment modalities have been suggested to manage patients with significant pelvic ring injuries and ongoing hemodynamic instability: Angio-embolization addressing arterial bleeding vs. pelvic packing, mainly controlling venous hemorrhage and bleeding from the spongious pelvic fracture site.

Our group previously pointed out that these two treatment modalities “*are not antagonistic but rather should be seen as complementary modalities*” (26, 27). In our own experience, signs of ongoing hemorrhage after mechanical pelvic stabilization and pre-peritoneal pelvic packing indicate the requirement for a postoperative pelvic angiography. Embolization of remaining arterial bleeding can then be performed on the way from the operating room to the Intensive Care Unit (ICU) in a patient with a more stabilized hemodynamic status. This concept of pre-peritoneal pelvic packing, external fixation of the pelvic ring injury and secondary angio-embolization is not new and has previously been described. Burlew et al. (28) reported on 75 patients with severe pelvic fractures and life-threatening hemorrhage, which underwent pelvic packing and external pelvic fixation. After surgery, a total of 10 patients (13%) successfully underwent angio-embolization; none of these patients died due to pelvic bleeding. The authors concluded that “*angio-embolization should be seen as a complementary procedure for life-threatening hemorrhage control”* following surgical pelvic packing and external fixation.

The goal of the present analysis is to assess the incidence of the need of postoperative angio-embolization after mechanical stabilization of the pelvic ring injury and direct pre-peritoneal pelvic packing. The sources of ongoing hemorrhage after surgical pelvic intervention and the patient's outcome are evaluated. We hypothesized that a secondary angio-embolization is effective in controlling persistent pelvic bleeding and will be required in a minority of patients following surgical damage control procedures.

## Methods

After approval by the Institutional Review Board, we performed a single center, retrospective observational cohort study, reviewing all severely injured trauma patients with pelvic ring injuries which were admitted to our level I trauma center from January 1, 2010 to December 31, 2019. As part of our participation in the German Trauma Registry DGU® and as previously described (29, 30), all data was prospectively documented using a computer-based online documentation tool. Patients with pelvic ring injuries were identified using the Abbreviated Injury Scale (AIS) code 8561xx. Patients requiring immediate mechanical stabilization of their pelvic ring injury and pre-peritoneal pelvic packing were further analyzed. Other inclusion criteria were blunt trauma, primary admission and age ≥ 18 years. Patients were excluded if they died in the shock room or if they did not receive any type of damage control procedure (supra-acetabular external fixator, pelvic C-clamp, pre-peritoneal pelvic packing) for their pelvic ring injury.

The following variables were extracted from our database and from the patient's electronic file [X-rays and computed tomography (CT) scan, operation report, discharge summary]: pelvic fracture pattern (AO classification), Injury Severity Score (ISS), AIS score for each body region (head, chest, abdomen, extremity), acute management of the pelvic ring injury on day 0, age, gender, first values of blood pressure and pulse rate on admission, and outcome (blood transfusion requirement, ICU length of stay, mortality).

Further variables abstracted included time to surgical intervention (mechanical pelvic stabilization and pelvic packing), time to postoperative angio-embolization and the time required for angio-embolization. The bleeding sources noted during the angio-embolization were extracted from the procedure report of the interventional radiologist.

Our emergency department treatment algorithm for patients with severe pelvic fractures has been extensively described previously (26, 27, 31). In brief, immediately after arrival, a mechanical stabilization of the pelvic ring injury is performed using a pelvic binder if it has not been done in the pre-hospital setting. Patients with a systolic blood pressure of <90 mmHg despite volume management and transfusion of packed red blood cells (PRBC) are classified as “non-responder.” In these patients, urgent surgical exploration, direct pre-peritoneal pelvic packing and mechanical stabilization of the pelvis using a pelvic C-clamp and/or an anterior supra-acetabular external fixator are carried out; diagnostic procedures including a polytrauma CT scan are postponed until hemodynamic stability has been achieved. During direct pre-peritoneal pelvic packing, associated intraabdominal, thoracic, and extremity injuries are simultaneously assessed and are treated according to damage control principles.

Direct pre-peritoneal pelvic packing is performed via a midline incision from the symphysis pubis extending cranially. The bladder is retracted laterally, the pelvic brim is palpated and a total of three laparotomy packs are placed below the pelvic brim. The first is placed as posteriorly as possible just below the sacroiliac joint, the second is placed just anterior to the first, and the third sponge is placed in the retropubic space deep lateral to the bladder. Afterwards, the contralateral side is packed identically. A second look procedure with removal or change of the pelvic packs is carried out 24–48 h after the initial surgery.

In case of clinical and/or laboratory signs of ongoing bleeding, such as a persistent requirement of volume resuscitation and PRBC transfusion, increasing lactate or base deficit values noted on blood gas analysis, an angiography is done, ideally before ICU admission. If contrast extravasation is seen in the angiography, a selective embolization of the bleeding vessels, using coils or foam, is directly undertaken. Any evidence of vessel spasm or an abrupt cut-off of a vessel are considered as signs of vascular injury and an embolization is likewise carried out. To assess the success of the angio-embolization procedure, the interventional radiologist routinely performs an additional contrast run after the final embolization to ensure complete hemostasis. If hemodynamic stability is achieved following angio-embolization, the diagnostic work-up, including CT scans and plain radiographs of extremity injuries, is completed if it has not been done before the emergency surgery. Thereafter, the patient is transferred to the ICU for further resuscitation.

For the present analysis, the patient cohort was divided into two groups: patients with angio-embolization following mechanical pelvic ring stabilization and direct pre-peritoneal pelvic packing and patients without angio-embolization after emergency surgery. Primary outcome parameters included transfusion requirement [amount of PRBC/fresh frozen plasma (FFP) from emergency department admission to ICU admission, total amount transfused during hospital stay], and in-hospital mortality rate.

### Statistical Analysis

Demographic and clinical characteristics comparing the two groups were evaluated using bivariate analysis. *P*-values for categorical variables were derived from the 2-sided Fisher's exact test and for continuous variables from the Mann–Whitney *U* test. Values are reported as mean ± standard deviation (SD) for continuous variables and as percentages for categorical variables. All analysis were performed using the Statistical Package for Social Sciences (SPSS for Mac), version 24.0 (SPSS Inc., Chicago, IL).

## Results

**Figure 1:**
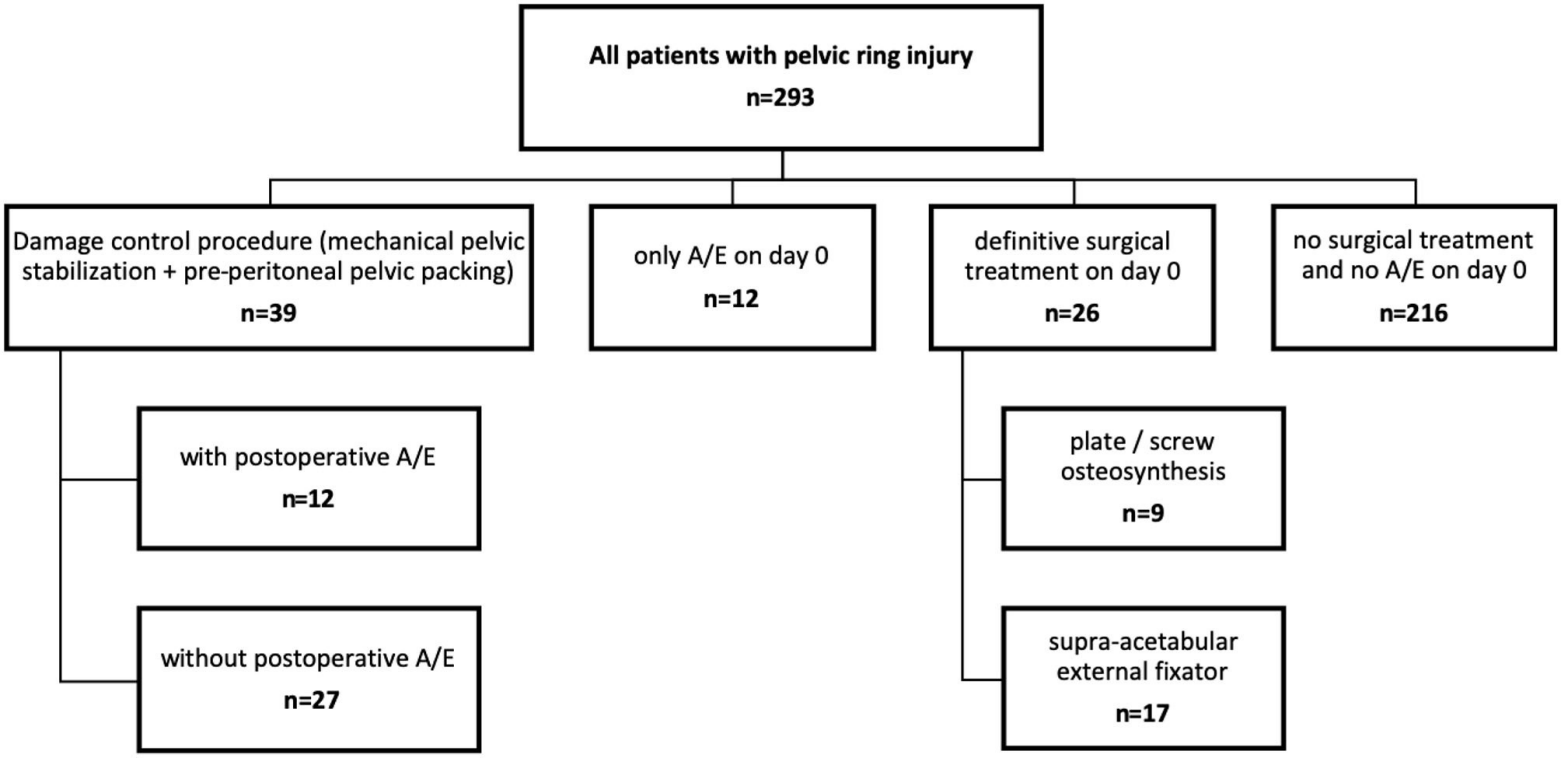
Flow chart demonstrating acute management of the pelvic ring injury on the day of admission. A/E, angio-embolization.

During the 10 year study period, a total of 293 patients with pelvic ring injuries were admitted. Of these, 39 patients (13.3%) required immediate mechanical pelvic stabilization and direct pre-peritoneal pelvic packing. A total of 12 patients (30.8% of the 39 patients) subsequently underwent postoperative angio-embolization due to persistent signs of ongoing bleeding (Figure 1).

Table 1 compares patients with and without angio-embolization following mechanical stabilization of the pelvic ring injury and direct pre-peritoneal pelvic packing. Patients which underwent postoperative angio-embolization had a significantly lower systolic blood pressure on admission, a higher ISS with a higher rate of severe head injuries (AIS head ≥3), received more PRBC and more FFP transfusion until ICU admission, and demonstrated a significantly higher lactate value on admission. The overall mortality rate, however, did not differ statistically significant between the two groups (without vs. with angio-embolization, 14.8 vs. 25.0%, *p* = 0.654).

**Table 1:** Characteristics and outcome of patients with and without postoperative angio-embolization.

	**All patients with damage control procedures *n* = 39**	**with A/E postoperative *n* = 12**	**without A/E postoperative *n* = 27**	***p*-Value**
Age (years), mean ± SD	46.5 ± 23.7	50.9 ± 20.2	44.6 ± 25.2	0.408
Male, % (*n*)	66.7% (26)	58.3% (7)	70.4% (19)	0.486
Systolic blood pressure on admission (mmHg), mean ± SD	95.7 ± 25.3	76.3 ± 15.1	104.4 ± 24.3	<0.001
RR sys <90 mmHg	35.9% (14)	75.0% (9)	18.5% (*5)*	0.001
Heart rate on admission	103.3 ± 25.0	110.6 ± 19.4	100.1 ± 26.8	0.065
GCS, mean ± SD	6.4 ± 5.1	6.0 ± 4.8	6.6 ± 5.3	0.822
ISS, mean ± SD	45.1 ± 14.0	52.8 ± 12.2	41.7 ± 13.6	0.018
AIS head ≥3, % (*n*)	46.2% (18)	83.3% (10)	29.6% (8)	0.002
AIS chest ≥3, % (*n*)	56.4% (22)	66.7% (8)	51.9% (14)	0.389
AIS abdomen ≥3, % (*n*)	48.7% (19)	58.3% (7)	44.4% (12)	0.423
AIS extremities ≥3, % (*n*)	97.4% (38)	100% (12)	96.3% (26)	1
**Laboratory values on admission**				
Hemoglobine (g/dL), mean ± SD	9.8 ± 2.5	8.6 ± 1.9	10.3 ± 2.6	0.052
Platelet count (×10^3^), mean ± SD	162.0 ± 56.9	140.5 ± 46.1	168.6 ± 59.0	0.270
Quick (%), mean ± SD	57.2 ± 16.9	52.1 ± 9.0	58.8 ± 18.6	0.397
aPTT (s), mean ± SD	41.3 ± 11.4	45.1 ± 10.6	39.9 ± 11.6	0.158
Base deficit (mmol/L), mean ± SD	5.21 ± 4.90	5.23 ± 8.04	5.20 ± 3.53	0.288
Lactate (mmol/L), mean ± SD	27.7 ± 19.2	40.8 ± 19.9	23.6 ± 17.4	0.020
**Outcome**				
PRBC until admission ICU, mean ± SD	12.1 ± 8.3	15.6 ± 4.2	10.6 ± 9.3	0.006
total PRBC during hospital stay, mean ± SD	16.3 ± 9.3	22.3 ± 5.8	13.6 ± 9.4	0.001
FFP until admission ICU, mean ± SD	9.6 ± 9.4	12.5 ± 4.0	8.4 ± 10.8	0.014
total FFP during hospital stay, mean ± SD	10.6 ± 11.4	13.1 ± 6.3	9.6 ± 13.0	0.042
In-hospital Mortality, % (*n*)	17.9% (7)	25.0% (3)	14.8% (4)	0.654

*A/E, angio-embolization; SD, standard deviation; RR sys, systolic blood pressure; GCS, Glasgow Coma Scale; ISS, Injury severity score; AIS, abbreviated injury scale; PRBC, packed red blood cells; FFP, fresh frozen plasma*.

Of the 12 patients with postoperative angio-embolization, 3 patients suffered a type B2 and 9 patients a type C pelvic ring injury (C1, *n* = 3; C2 *n* = 2; C4, *n* = 4).

The mean time from admission to surgical intervention was 52.8 ± 14.7 min (range 34–76 min). All 12 patients underwent mechanical pelvic stabilization (supra-acetabular external fixator, *n* = 12; pelvic C-clamp, *n* = 3) and direct pre-peritoneal pelvic packing (*n* = 12). A total of 10 patients required further damage control procedures including external fixators of extremities (*n* = 10), laparotomy to address intra-abdominal associated injuries (*n* = 5) and intracranial pressure monitoring and/or craniotomies or craniectomies for the traumatic brain injury (*n* = 7). The mean time from hospital admission to postoperative angio-embolization was 189.1 ± 55.5 min (range 111–289 min) and the mean time required for the angio-embolization procedure was 52.3 ± 28.3 min (range 21–124 min).

Table 2 describes the angiographically identified bleeding sources and the type of embolization performed. In one patients, two bleeding vessels were identified during angiography. While in 11 patients a selective embolization of the bleeding vessels was undertaken, in 1 patient an unilateral complete occlusion of the internal iliac artery had to be performed. The superior gluteal artery was the most often identified bleeding source (*n* = 6, 42.6%). In all 12 patients, the angio-embolization procedure was successfully performed achieving complete hemostasis in the pelvic region. No complications associated with the embolization, such as necrosis or ischemia of tissues occurred during the hospital stay.

**Table 2:** Bleeding sources and type of embolization in patients treated with mechanical pelvic stabilization and pelvic packing followed by angio-embolization.

**Bleeding source**	***n***	**Type of embolization**
Superior gluteal artery	6	Selective
Obturator artery	2^*^	Selective
Internal pudendal artery	2^*^	Selective
Unnamed branches of internal iliac artery	3	1× complete occlusion int. iliac artery, 2× selective

* *1 patient with two bleeding sources*.

The mean transfusion requirement of packed red blood cells until angio-embolization was 15.6 ± 4.2 (range 9–21 packs). After angio-embolization until 48 h after admission, a mean of 5.1 ± 2.1 packs of red blood cells (range 2–9 packs) had to be transfused. The second look procedure was performed within 27.9 ± 8.6 h after the first operation. In two patients the pelvis was repacked due to persistent oozing. No complications related to the packing, such as pelvic space infection, were observed.

The mean ICU length of stay was 17.9 ± 10.7 days (range 3–42 days). Overall, three patients died in the further hospital course; two patients due to multiple organ failure (days 7 and 12, respectively) and one patient due to the traumatic brain injury (day 3).

## Discussion

In the present study analyzing a 10-year period, approximately 13% of patients with pelvic ring injuries required emergent mechanical pelvic ring stabilization and direct pre-peritoneal pelvic packing due to hemodynamic instability. Of these patients, approximately 30% underwent post-operative angio-embolization to address ongoing arterial bleeders. The main finding is, that—by following the presented treatment algorithm—none of these highly unstable and critically injured pelvic ring injury patients (mean ISS of 53 points) died due to hemorrhage. Furthermore, we noted that the most often found bleeding artery after pelvic packing was the superior gluteal artery, which is difficult to surgically address.

In severe pelvic ring injuries, the bleeding source is more frequently of venous than arterial origin (90 vs. 10%). However, in pelvic trauma patients with hemodynamic instability, these numbers significantly change and a higher rate of arterial extravasation is observed in these cases. In the study by Eastridge et al. (7), 58.7% of patients with a severely unstable pelvic ring injuries and ongoing hemodynamic instability demonstrated an arterial vascular lesion. Similarly, Miller and colleagues reported a 67.9% rate of arterial bleeding in patients with ongoing hemodynamic compromise (12). However, although angio-embolization is clearly the treatment of choice in pelvic ring injury patients with arterial hemorrhage, numerous downsides of this procedure need to be considered, which—in our opinion—limit its safe use in the hemodynamically compromised trauma patient. First, angiography is known to possibly be a time-consuming procedure and simultaneous diagnostic or therapeutic interventions are not or only very limited possible in the angiography suite. Second, it necessitates the timely (24/7) availability of a highly skilled and trained interventional radiologist, but also associated technical assistants. Furthermore, the entire procedure needs to be set up quickly and without significant delay between end of the shock room treatment and start of angiography. This time and availability requirement has been shown to be one, if not the most important drawback of this procedure in critical situation with hemodynamically unstable patients. Numerous studies have highlighted that considerable time delays, ranging from 50 min up to 5.5 h, exist in performing the angiography procedure, what might obviously not be tolerated by a patient with hemodynamic instability (4, 10, 13). In a multicenter study, including 11 major trauma center in Australia and New Zealand and describing the treatment practice in hemodynamically compromised patients with severe pelvic ring injuries, only 14.7% of the angiography procedures were started within 90 min of hospital admission (32). Considering the time interval between admission and start of surgical procedure in our patients, a mean time to surgery of 52.8 ± 14.7 min, ranging from 34 to 76 min, was found, which accurately reflects the importance of achieving fast hemorrhage control.

Identifying whether venous or arterial bleeding is the major source responsible for the hemodynamic instability is not possible in the acute resuscitation phase. Venous bleeding from the fractured bony surface and from the pre-sacral and pre-vesical plexus is almost always present in significant pelvic ring injuries. Therefore, considering the previously listed drawbacks of angiography, mechanical stabilization of the pelvic fracture and pelvic packing is the first line of treatment in our management algorithm. Simultaneously, other necessary damage control techniques for concomitant head, chest, abdominal, and/or extremity injuries are applied. In case of ongoing hypotension and/or ongoing transfusion requirement following mechanical pelvic stabilization and pelvic packing, angiography and embolization of persistent arterial bleeders is performed. The intention by following this sequence of treatment modalities (surgery as first line, angio-embolization as second line of treatment) in these highly unstable patient, is to have a hemodynamically at least transient stable patient in the angiography suite. Additionally, surgery as first line procedure may “buy” time for setting up the emergent pelvic angiography. Our management algorithm is in line with previously reported protocols for patients with pelvic ring injuries (22, 28, 33–35). In the study by Burlew and coauthors, 13% of the patients underwent secondary angio-embolization. The mortality rate in this high-risk patient group was 21% with only 2% (*n* = 3) death cases due to acute bleeding (33). Magnone et al. (22) recently published a prospective validation of a pre-peritoneal pelvic packing protocol for hemodynamically unstable pelvic trauma patients. Similar to the previously mentioned study, the authors concluded that pelvic packing was a quick and effective method in the treatment of these severely injured patients. However, in contrast to our study, not all patients received a mechanical stabilization of the pelvic ring injury, which—in our opinion—is essential in order to provide sufficient abutment for the pelvic packs. Lastly, hybrid operating room systems have to be mentioned. In multiple studies, hybrid operating rooms have been shown to improve the management and workflow in patients with severe pelvic ring injuries (36–38). Ito and colleagues recently demonstrated that the time from admission to angio-embolization was significantly shorter in the hybrid operating room group. At our institution (and likely in the vast majority of trauma centers worldwide), hybrid operating room systems are currently not available, however, it would clearly pose a significant advantage in the management of these patients (38).

The most commonly reported arteries that require embolization in patients with pelvic ring injuries are the internal iliac artery in approximately 67%, unnamed branches of the internal iliac artery (17%), the superior gluteal artery (4%), the obturator artery (4%), and the internal pudendal artery (3%) (39). Multiple studies have addressed the association between pelvic fracture pattern and hemorrhage (10, 40–42). Due to the proximity, the superior gluteal artery is at risk in fractures involving the sciatic notch (39). Fractures of the pubic rami are associated with obturator vessel laceration and disruption of the sacroiliac joint with hemorrhage from gluteal and hypogastric branches (43, 44). To the best of our knowledge, the present study is one of the few investigations that specifically evaluates the angiographically identified sources of arterial bleeding following mechanical stabilization and surgical exploration and packing of the pelvic ring injury. In almost half of the cases, the superior gluteal artery was identified as the ongoing bleeder, which is, due to its anatomic course, difficult to surgically address. Contrary to the other branches of the internal iliac artery, the superior gluteal artery exits the small pelvis through the greater sciatic foramen. Therefore, and due to its dorsal position outside the pelvis, it is highly challenging—if not impossible—to perform surgical hemostasis or to pack the artery sufficiently. Its course outside the pelvis likewise exposes the superior gluteal artery to the risk of laceration when a percutaneous sacroiliac screw insertion is performed (45, 46). The obturator artery, on the other hand, which was found in 2 cases as the bleeding source, may be more accessible for surgical hemostasis. However, retraction of the vessel stump or vasospasm may complicate the identification of its laceration. Embolization was in all but one case performed in a selective way. A complete unilateral occlusion of the internal iliac artery was necessary in one case to achieve sufficient hemostasis; however, no complications associated with the complete occlusion were observed in the further hospital course.

The present study has several limitations, the most important being the retrospective study design. Furthermore, only a very selected group of pelvic ring injury patients was examined in this investigation. Patients not receiving damage control interventions for their pelvic ring injury on the day of admission and deaths in the shockroom were excluded from the analysis, introducing a selection bias. As a consequence, the reported mortality rate in the present study cannot be compared with mortality rates from studies looking at all patients with severe pelvic ring injuries. Additionally, due to our strict inclusion criteria, the number of patients analyzed in our study is low. However, it is important to notice, that these patients are a highly critical and severely injured subgroup of pelvic ring injury patients (mean ISS of 53 points), that are—even in high-volume trauma centers as ours—not frequently seen. Finally, the study periods spans over 10 years, in which changes and improvements in volume and transfusion management have occurred. However, with regards to our management algorithm for patients with severe pelvic ring injuries, no significant changes were introduced in our clinic during the study period. As we previously published, interventional emergency embolization for severe pelvic ring fractures with arterial bleeding is an integral part in our treatment algorithm since many years (13).

## Conclusion

According to our management protocol, all hemodynamically unstable patients with severe pelvic ring injuries were directly transferred to the operating room, where mechanical stabilization of the pelvic ring, direct pre-peritoneal pelvic packing, and, in approximately one third of the patients, a subsequent angio-embolization was carried out. This sequence of treatment modalities resulted in a complete hemostasis of the pelvic hemorrhage in all of the examined cases. The superior gluteal artery, which is—due to its position and course in the pelvis—difficult to access, was the most often found ongoing arterial bleeder following surgical exploration and pelvic packing, further highlighting the importance of the secondary angio-embolization.

## Data Availability Statement

The raw data supporting the conclusions of this article will be made available by the authors, without undue reservation.

## Ethics Statement

The studies involving human participants were reviewed and approved by the Institutional Review Board University Hospital Frankfurt. Written informed consent for participation was not required for this study in accordance with the national legislation and the institutional requirements.

## Author Contributions

TL, PS, KE, and IM participated in research design, data analysis and wrote the paper. CN and MJ participated in research design and data analysis. All authors contributed to the article and approved the submitted version.

## Conflict of Interest

The authors declare that the research was conducted in the absence of any commercial or financial relationships that could be construed as a potential conflict of interest.
